# Oral Gabapentin Versus Rectal Diclofenac for Postoperative Analgesia

**DOI:** 10.5812/atr.9855

**Published:** 2013-02-01

**Authors:** Smita Prakash

**Affiliations:** 1 Department of Anaesthesia and Intensive Care,Vardhman Mahavir Medical College and Safdarjang Hospital, New Delhi, India

**Keywords:** Analgesia, Diclofenac, Gabapentin, Postoperative, Tonsillectomy

*Dear Editor,*

I read with interest the article by Mogadam et al. (1) in which the authors have compared the analgesic efficacy of gabapentin and diclofenac in patients undergoing tonsillectomy. They concluded that both gabapentin and diclofenac reduced postoperative pain and opioid consumption without side effects. Four additional points deserve commentary and clarification. Gabapentin is being increasingly used as a multimodal perioperative drug because of its ability to produce analgesia, anxiolysis and sedation. Pre-treatment with gabapentin has been shown to allay preoperative anxiety (2). In this study, patients received atropine and alfentanyl as premedication. It would benefit the readers to know if patients who received gabapentin preoperatively displayed greater anxiolysis compared with those who received placebo or rectal diclofenac. Gabapentin 800 mg administered 1 - 2 h preoperatively attenuates the increase in blood pressure in the first 10 min following laryngoscopy and intubation (3). Was there any significant difference in hemodynamic parameters between the three groups during laryngoscopy and intubation or during the intraoperative period? Gabapentin decreases analgesic consumption and opioid related adverse effects; however, this is at the expense of increased sedation and dizziness (4). Somnolence is a side effect observed in 15.2% of patients (5). It would be interesting to know whether there was any difference in the degree of sedation in their patients during the postoperative period. Also, was there any significant difference in intraoperative isoflurane requirement within the three groups? Diclofenac rectal suppository is absorbed in 30 - 60 min and achieves T max after 50 min of insertion. It offers a simple means to administer the drug, equalling the analgesic efficacy of the oral preparation (6). It bypasses the enteric system thus eliminating several gastrointestinal adverse effects of the drug. While Taj et al. (7) found that all children in their study accepted rectally administered drug, Tolksdorf et al. (8) found that oral midazolam was better accepted than rectal midazolam. Did the authors find the rectal administration of diclofenac during the preoperative period to be acceptable to patients or would the patients have preferred the oral route of premedication?
